# Parent-Mediated Interventions for Infants under 24 Months at Risk for Autism Spectrum Disorder: A Systematic Review of Randomized Controlled Trials

**DOI:** 10.1007/s10803-021-05148-9

**Published:** 2021-07-08

**Authors:** Mei L. Law, Jatinder Singh, Mathilde Mastroianni, Paramala Santosh

**Affiliations:** 1 Department of Child and Adolescent Psychiatry, Institute of Psychology, Psychiatry and Neuroscience, King’s College London, Addiction Sciences Building, 1-4 Windsor Walk, Denmark Hill, London, London, SE5 8BB UK; 2 Centre for Interventional Paediatric Psychopharmacology and Rare Diseases, South London and Maudsley NHS Foundation Trust, London, UK

**Keywords:** Autism spectrum disorder, At risk, Infants, Parent, Interventions, Systematic review

## Abstract

Prodromal symptoms of Autism Spectrum Disorder (ASD) have been detected within the first year of life. This review evaluated evidence from randomized controlled trials (RCTs) of parent-mediated interventions for infants under 24 months who are at risk for ASD. Electronic databases, including grey literature, were searched up till November 2019. Seven RCTs were identified. There was substantial heterogeneity in recruitment, outcome measures and effect size calculations. Interventions did not reduce the risk of later ASD diagnosis and post-intervention effects on infant outcomes were inconsistent, with five studies reporting significant improvements across both treatment and control groups. Moderate level of evidence of intervention effects on parental interaction skills and the small number of RCTs, and significant limitations restrict generalizability across studies.

**Supplementary Information:**

The online version contains supplementary material available at 10.1007/s10803-021-05148-9.

## Introduction

Autism Spectrum Disorder (ASD) is a neurodevelopmental disorder characterized by social communication deficits and restricted, repetitive patterns of behaviour (American Psychiatric Association, 2013). With a prevalence of one in 59 children (Baio et al., 2018), the burden of ASD is also one of the highest among mental disorders in young children (Baxter et al., 2014). ASD manifests in early childhood and persists into adulthood, creating multiple challenges for children and families. Besides social communication and behavioural deficits, children with ASD also face difficulties at life transitions and have poorer outcomes as adults (Lai et al., 2014). The average age of formal autism diagnosis is around 4 to 5 years old (Crane et al., 2016; Zuckerman et al., 2017). Delays between first concerns and formal diagnosis are well-reported, at an average delay of 2.2 years in the US (Zuckerman et al., 2017) and 3.5 years in the UK (Crane et al., 2016). Early parental concerns about infants’ or toddlers’ atypical development may receive little attention until formal ASD diagnosis (Freuler et al., 2014), which is less likely to be confirmed under 2 years old (Crane et al., 2016; Pasco, 2018). Early detection (or diagnosis) of ASD by primary care professionals is crucial to expedite referrals to early intervention services (Dunlap, 2019). Longitudinal studies have shown that early language and attentional skills predicted social functioning outcomes in adults with ASD (Gillespie-Lynch et al., 2012), illustrating potential targets for early interventions. In theory, early interventions in ASD may have the potential to alter later developmental outcomes if implemented within the first 2 years of life, which is a critical period of brain development (Botteron, 2019; Dunlap, 2019). Recent research suggests that interventions can be initiated before the emergence of core ASD symptoms. Behavioural risk markers (or prodromal symptoms) have been detected as early as 12 months of age, which predicted ASD diagnoses at 36 months (Lai et al., 2014; Sacrey et al., 2015; Webb et al., 2014). Theoretically, interventions that target prodromal symptoms can influence critical periods of brain development and alter the manifestation of ASD symptoms (Dawson, 2008).

General guidance provided by the National Institute for Health and Care Excellence (NICE) in the UK and the Centers for Disease Control and prevention (CDC) in the US include interventions to manage symptoms of ASD and associated difficulties in children and young people (Centers for Disease Control & Prevention, 2020; National Institute for Health & Care Excellence, 2013). Early interventions in ASD encompass a wide variety of approaches with different targets for treatment, including behavioural components, developmental components, multicomponent methods involving both behavioural and developmental approaches, a range of different communication techniques and also technology-based approaches (French & Kennedy, 2018). Intervention programmes for toddlers and pre-schoolers typically incorporate facilitation of parent–child interactions, behavioural modifications, and changes to learning environments (Pasco, 2018). Both NICE and CDC guidance highlight essential significant adjustments to the child’s social and physical environment in interventions (Centers for Disease Control & Prevention, 2020; National Institute for Health & Care Excellence, 2013). The implementation of these changes would require parental involvement, with parents trained as co-therapists in interventions to provide consistency and support the transfer of children’s skills from therapeutic and school settings to their family homes (McConachie & Diggle, 2007; Pasco, 2018). Considering this, interventions initiated in the first 2 years of life would benefit from active parental involvement. Parent-mediated interventions might potentially target atypical development at a crucial stage of brain development for infants at risk for ASD (Webb et al., 2014). Some studies have reported beneficial effects for parents, such as increased parenting skills and reduction of maternal depression, which subsequently impact the development of the affected child and their siblings (Lai et al., 2014; McConachie & Diggle, 2007). Aside from that, mobilizing parents in implementing interventions at home can help overcome the challenges in accessing community services (Dowden, 2018; Sacrey et al., 2015).

### Recent Reviews

Based on a scoping review, at least ten systematic reviews and meta-analyses have been published in the field of ASD interventions from infancy up to early childhood (see summary in Table 1). Three reviews were focused on toddlers under 36 months old. Of these, a systematic review of 26 studies (Morgan et al., 2014) and meta-analysis of 34 single-subject studies (Debodinance et al., 2017) reported that most interventions had significant caregiver involvement, with positive treatment effects in most outcomes especially for interventions carried out at home. Both studies reported overall gains in social communication outcomes while Debodinance et al. (2017) additionally reported positive behavioural outcomes. A systematic review of nine studies (including RCT, case-series and quasi-experimental studies) (Bradshaw et al., 2015) with infants under 24 months reported that most interventions in this age group were parent-mediated, with positive gains in infant outcomes including improvements in language, communication and behaviour. This review also found positive parental outcomes such as high feasibility, lower stress and increased parental skills. Four reviews focused predominantly on parent-mediated interventions; these included children between 1 to 6 years old with formal ASD diagnoses (Hong et al., 2018; McConachie & Diggle, 2007; Nevill et al., 2018; Oono et al., 2013). Two other reviews included all types of interventions, with all children up to 6 years old diagnosed with or at risk for ASD (French & Kennedy, 2018; Reichow et al., 2012). Inconclusive results were reported in these reviews, ranging from no effects to positive effects on parent–child interactions and moderate reduction in ASD symptom severity. However, a review of home-based intensive behavioural interventions found that in one study, intervention did not improve parental stress, anxiety or depression levels, with higher reports of depression in fathers of children in the intervention groups (Reichow et al., 2012). Lastly, a review of observational studies in infants under 24 months with familial risk of ASD showed differences in parent-infant interaction (Wan et al., 2018); although not based on interventions, this review was included in the summary due to the relevance of the target population.

**Table 1:** Summary of systematic reviews and meta-analyses on ASD interventions for children up to 6 years old, ordered by year of publication

Reference and Year	Type of review	No. of studies	Age group	ASD status	Studies	Interventions
McConachie and Diggle (2007)	SR	71	1 year to 6 years 11 months	Diagnosed	Groups only with control or comparison groups	Parent-implemented or significant focus on parent-implementation
Reichow et al.,(2012)	SR and MA	5	Under 6 years	Diagnosed	RCT, quasi-RCT, CCT with control condition	All behaviour-focused interventions, no information on parent involvement
Oono et al., (2013)	SR	17	1 year to 6 years 11 months	Diagnosed	RCT	Parent-mediated only
Morgan et al., (2014)	SR	26	Under 36 months	Diagnosed and at risk	Single-subject and groups with control mechanism	All social-communication interventions, delivered jointly by professional and caregiver
Bradshaw et al., (2015)	SR	9	Under 24 months	Diagnosed and at risk	Experimental designs; single-case, RCT, quasi-experimental	All interventions; 8 parent-mediated and 1 therapist-delivered
Debodinance et al., (2017)	MA	34	Under 36 months	Diagnosed and at risk	Experimental designs; single-subject only	All psychosocial interventions, most with 50% caregiver involvement
French and Kennedy (2018)	SR	48	Up to 6 years (72 months)	Diagnosed and at risk	RCT	All interventions, 27 therapist-delivered with parent-training, 7 parent training, 14 therapist-delivered only
Hong et al., (2018)	SR and MA	34	Unspecified—“Children”	Diagnosed	Single-case and groups, with time-series data for individual participants	Caregiver-delivered
Nevill et al., (2018)	MA	19	1 year to 6 years	Diagnosed	RCT	Parent-mediated only
Wan et al., (2018)	SR	15	Under 24 months	At risk – siblings of children with ASD	Studies with control or comparison groups	Non-interventional, this review is based on observational studies of parent–infant interaction

*ASD* Autism Spectrum Disorder, *CCT* clinical control trials, *MA* meta-analysis, *RCT* randomized control trials, *SR* systematic review

### Objective

Given the potential of parent-mediated interventions and the possibility of targeting prodromal symptoms (Lai et al., 2014; Sacrey et al., 2015; Webb et al., 2014), there is a gap in research on reviews of interventional studies in infants and toddlers who are *at risk for* ASD only. Increasing numbers of randomized controlled trials (RCTs) suggest increased rigour of early intervention research in this area (French & Kennedy, 2018). This warrants an updated review focused predominantly on the impact of parent-mediated interventions in this age and diagnostic group. The overall aim of this study is to systematically review evidence from RCTs of parent-mediated interventions for infants and toddlers under 24 months of age (hereafter, “infants”) who are at risk for ASD. Guided by the PICOS elements, the objective of this review is to evaluate studies with the following criteria; Participants (P): infants under 24 months of age who are at risk for ASD, Interventions (I): all interventions targeted at reducing or improving outcomes related to ASD, implemented or mediated by parents only, Comparison (C): control groups are required, such that participants need to be randomly assigned to intervention or control conditions, Outcomes (O): both infant and parental outcomes are evaluated, Study design (S): only randomised controlled trials (RCTs) are included.

## Methods

This review was registered with the International Prospective Register of Systematic Reviews (PROSPERO) (Reg. Number: CRD42018117351). Parent-mediated interventions focused primarily on at-risk infants are relatively novel in the field of early autism intervention (Bradshaw et al., 2015). This review considered all models of parent-mediated interventions implemented by researchers, but strict eligibility criteria were imposed for infants’ age and ASD risk status. The PRISMA checklist (Moher et al., 2009) was used to guide the content of this review.

### Search Strategy

A systematic search was conducted on the following databases: PsycINFO, PsycARTICLES Full Text, Global Health, MEDLINE® and Epub Ahead of Print, In-Process & Other Non-Indexed Citations and Daily, EMBASE, Web of Science Core Collection, and ProQuest Dissertations and Theses Global up to 4 November 2019. No date limit was imposed on all searches, i.e. all databases were searched ‘from inception’. Due to the focus on narrow age range and diagnostic classification, the search strategy was intentionally inclusive of all interventions in the first instance. The search terms also included ‘autism-spectrum condition’ or ‘asc’ to identify studies which may have used this term, reflecting increasing preference for less-stigmatising language^1^ in research (Baron-Cohen et al., 2009).The search terms used were: interven* AND infant* or baby or babies or “young child*” or toddler* AND risk adj2 (autis* or asc or ASD or pdd or “pervasive developmental disorder*”) (see Supplementary Material for database variations). Searches were conducted on two occasions by two independent reviewers (MLL and MM): 4 November 2019 and 16 May 2020, where the second search was performed to capture new publications between these dates.

### Inclusion Criteria

Studies were included if: (1) the mean age of infants were under 24 months (24 months and younger), (2) infants’ ASD risk status were clearly cited by authors, (3) interventions were implemented by parents only, and (4) studies were RCTs.

### Exclusion Criteria

Studies were excluded if: (1) any infants were diagnosed with ASD prior to study, (2) the results were based on the same group of infants, (3) they were not in English and no translation was available.

### Article Selection

Screening and selection of articles were conducted independently by MLL and MM based on the eligibility criteria specified. After the removal of duplicates, studies were screened by title, and then by abstract. After that, full-text studies were read and selected. Final article selections were then compared for reliability. Finally, references of eligible studies were screened, and authors of pilot studies were contacted for follow-up studies.

### Quality Assessment: Cochrane’s Risk of Bias Criteria

This review assessed risk of bias in studies using the Cochrane Collaboration’s tool (Higgins et al., 2011), which recommends the evaluation of studies based on seven key domains: sequence generation, allocation concealment, blinding of participants/personnel, blinding of outcome assessment, incomplete outcome data, selective reporting, and other bias. In this review, the authors discussed the need to specify “other bias”, and agreed to expand this domain to separately evaluate the reporting of effect sizes and declaration of conflicts of interest in studies. Risk of bias appraisals were performed by MLL and MM independently, where ratings of low, unclear or high were assigned. Interrater reliability was assessed for every domain and overall judgement of each study. Disagreements were either ‘substantial’ (i.e., one reviewer rated a study low and another reviewer rated it high) or ‘moderate’ (i.e., one reviewer rated a study unclear and another rated it low or high), to discern types of disagreement (Hoy et al., 2012).

### Interrater Reliability

Using SPSS Version 26, percentage agreement and Cohen’s Kappa (κ) were calculated for the assessment of reliability in article selection and risk of bias ratings. In some instances, Cohen’s Kappa was indeterminate as ratings within a domain were constant. Percentage raw agreement were used in these cases. Final decisions on article selections and risk of bias were determined by consensus.

### Data extraction

Data extraction from included studies was performed by MLL and reviewed by all authors. Data extraction was also guided by PICOS (Moher et al., 2009): Participants, Interventions, Control, Outcomes and Study Design. These categories were expanded to describe sampling methods, sample sizes, determinants of ASD risk, type and intensity of interventions, type of controls, infants’ outcomes and parental outcomes. As part of the inclusion criteria, all Study Designs are RCTs as standard and this item was not included in data extraction.

## Results

**Fig. 1:**
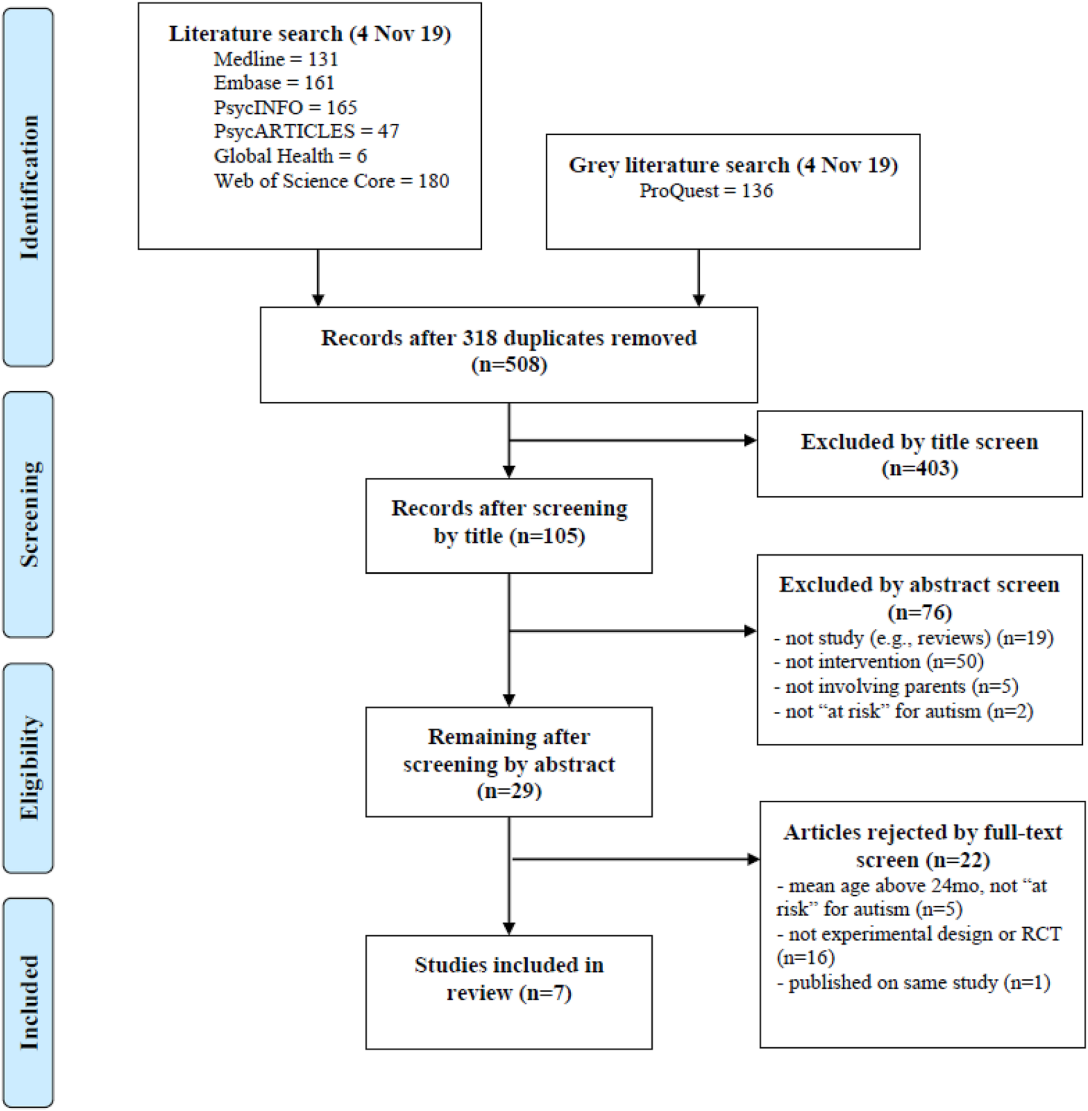
PRISMA flow diagram (Moher et al., 2009)

A PRISMA flow diagram depicting the screening procedure is displayed in Fig. 1. A total of 508 unique studies were found on electronic databases and grey literature. Among these, 403 articles were excluded by title screen and 76 articles by abstract screen. MLL had selected seven studies (out of 29 full-text studies read), while MM had selected ten studies (out of 24 full-text studies read), resulting with an overall agreement on seven studies (70%). Disagreement on the remaining three studies were discussed and agreed by consensus. Of these, one study (Kasari et al., 2015) was excluded as participants were ‘toddlers *with* ASD’, while another study (Fox, 2017) (unpublished dissertation) was excluded as the mean age of participants was 32.8 months, with nine of ten participants having confirmed diagnosis of ASD. Finally, one study (Green et al., 2017) published longitudinal outcomes and was based on the same group of infants of a study already included in the final selection (Green et al., 2015). As this is not considered an independent study for the purposes of this review, it was not formally excluded and its findings were synthesized with the first publication. Authors of two pilot studies (Rogers et al., 2014; Steiner et al., 2013) were contacted through email to confirm that no follow-up studies had been conducted.

Seven RCTs were included in the final review. Characteristics (see Table 2) and outcomes (see Table 3) of studies were extracted and displayed according to publication year. Studies were published between 2012 and 2019; five in the US (Baranek et al., 2015; Jones et al., 2017; Kasari et al., 2014; Rogers et al., 2012; Watson et al., 2017), one in UK (Green et al., 2015) and one in Australia (Whitehouse et al., 2019). The UK study (Green et al., 2015) published longitudinal follow-up results (Green et al., 2017), which were synthesized within the findings.

**Table 2:** Characteristics of studies

Study	Sampling location	Number and Age in months	Determinant of autism risk	Intervention type and intensity	Goals	Control		Assessment points	Risk of Bias
Rogers et al., (2012)	USA PD, Regional Centers, University autism clinics, Research Programs	Intervention = 49 (Mean age = 21.02) Control = 49 (Mean age = 20.94) Age range = 12–24 months	ITC (all children) ESAT (12–15 months olds) M-CHAT (16–24 month olds)	Parent-delivered Early Start Denver Model (P-ESDM) 12 weeks 12 sessions 60 min Once a week Sessions and assessments conducted at university clinics	Gains in social communication and Mullen developmental quotients, reduced core autism symptoms	Community intervention		Baseline, Post-intervention (12 weeks)	Unclear
Kasari et al., (2014)	USA PD, EI Program, Regional Centers, Autism Evaluation Clinics	Intervention = 32 (Mean age = 22.56) Control = 34 (Mean age = 22.19) Age range = 15–31 months	M-CHAT CSBS DP	Focused Playtime Intervention (FPI) 12 weeks 12 sessions 90 min Once a week Sessions conducted at home, assessments conducted in university laboratories	Promote coordinated toy play between parent and child, improve parental responsiveness, and improve child's communication and language, including joint attention and Mullen developmental quotients	Monitoring: Four 90-min in-home sessions to promote social and emotional competence, and addressing challenging behaviours		Baseline, Post-intervention (3 months), Follow-up (12 months after baseline)	Low
Baranek et al., (2015)	USA Community sample from birth registry records	Intervention = 11 (Mean age = 15.22) Control = 5 (Mean age = 15.6) Age range = 13–17 months	First Year Inventory (FYI) (autism risk) AOSI/ADOS-T, MSEL, CSBS, MCDI, ITSEA, SPA and SEQ (eligibility in study)	Adapted Responsive Teaching (ART) 6–8 months 20–39 contacts (mean = 33.5) 1st 6 weeks: 2 home sessions per week 2nd 6 weeks: 1 home session and 1 phone call per week Last 12 weeks: 1 home session per week Sessions conducted at home, assessments conducted in assessment suite near university	Improve infants' pivotal behaviours in social communication and sensory-regulatory functions	Referral to early intervention and monitoring (REIM), may receive community EI services		Baseline, Post-intervention (8–9 months), Follow-up (18–19 months after baseline)	Unclear
Green et al., 2015	UK British Autism Study of Infant Siblings (BASIS)	Intervention = 28 (Mean age = 276.58 days) Control = 26 (Mean age = 267.14 days) Age range = 7–10 months	Older sibling with autism	British Autism Study of Infant Siblings, Video Interaction for Promoting Positive Parenting (iBASIS-VIPP) 5 months 6–12 sessions (Mean = 9.5) 2 h Weekly to fortnightly sessions Sessions conducted at home, assessments conducted at laboratories	Modify early risk markers for ASD, including atypical interaction (infant attention to parent) and ASD behaviour	No treatment		Baseline, Post-intervention (6 months), *Follow-up 1 (18 months after baseline)* *Follow-up 2 *(*30 months after baseline*) (Green et al., 2017*)*	Low
Jones et al., (2017)	Location and recruitment not reported	Intervention = 19 (Mean age = 192.3 days) Control = 14 high risk A+M (Mean age 194.4 days), 150 normative controls Age range = 6 months	Older sibling with autism (diagnosis confirmed with ADI-R)	Promoting First Relationships (PFR) 10 weeks 60–85 min Once a week Sessions conducted at home, location of assessments not reported	Stimulate neural systems associated with social interaction, promote infant's attention and response to social partner	High risk group for Assessment and Monitoring (A+M), Normative controls to provide data on developmental norms		Baseline, Post-intervention (6 months), Follow-up (12 months after baseline)	High
Watson et al., (2017)	USA Community sample; FYI mailed to catchment area of six central counties of North Carolina	Intervention = 45 (Mean age = 13.8) Control = 42 (Mean age = 13.7) Age range = 13–16 months	First Year Inventory (FYI)	Adapted Responsive Teaching (ART) 6–8 months 30 in-home sessions and 6 contacts via phone/email 1st 6 weeks: 2 home sessions/week 2nd 6 weeks: 1 home session and 1 phone call/email/week Last 12 weeks: 1 home session/week Sessions conducted at home, assessments conducted at community-based facilities	Improve social-communication and sensory-regulatory functions, developmental and adaptive skills, and reduce severity of ASD symptoms	Referral to EI and Monitoring (REIM), may receive community EI services		Baseline, Post-intervention (~ 9 months)	Low
Whitehouse et al., (2019)	Australia Government service for children with developmental delays, Community maternity and child health nurses	Intervention = 50 (Mean age = 12.4) Control = 53 (Mean age = 12.38) Age range = 9–14 months	Social Attention and Communication Surveillance-Revised (SACS-R)	British Autism Study of Infant Siblings, Video Interaction for Promoting Positive Parenting (iBASIS-VIPP) 5 months 8–10 sessions Fortnightly sessions Sessions conducted at home, assessments conducted at university laboratories	Reduce severity of ASD symptoms, increase quality of parent–child interactions, and improve infant communication and social skills at treatment endpoint/follow-up	Community treatment-as-usual (TAU)		Baseline, Post-intervention (6 months) *Follow-ups are planned at 12 months and 18 months after baseline (pending)*	Low

*ADI-R* Autism Diagnostic Interview-Revised, *ADOS-T* Autism Diagnostic Observational Scale Toddler, *A+M* Assessment and Monitoring, *AOSI* Autism Observation Scale for Infants, *ASD* Autism Spectrum Disorder, *CSBS DP* Communication and Symbolic Behaviour Scales Developmental Profile, *EI* Early Intervention, *ESAT* Early Screening of Autistic Traits Questionnaire, *FYI* First Year Inventory, *ITC* Infant Toddler Checklist, *ITSEA* Infant-Toddler Social Emotional Assessment, *MCDI* MacArthur-Bates Communicative Development Inventory, *M-CHAT* Modified Checklist for Autism in Toddlers, *MSEL* Mullen Scales of Early Learning, *Mullen* Mullen Scales of Early Learning, *PD* Paediatricians, *SEQ* Sensory Experiences Questionnaire, *SPA* Sensory Processing Assessment, *TAU* Treatment as Usual

**Table 3:** Reported outcomes in studies

Study	Infant outcome	Diagnostic outcome	Parent outcome
Rogers et al., (2012)	No difference between P-ESDM and community treatment on child outcomes; significantly improved outcomes are listed below. At post-test, community group received more intervention hours than P-ESDM group 1. Developmental Quotient (Mullen): Improvement in Mullen DQ in both P-ESDM (d = 0.44) and community (d = 0.37) groups. Similarly, improvement in Verbal DQ in P-ESDM (Cohen's d = 0.56) and community (d = 0.53) groups. Combined groups, toddlers with younger age showed greater increase in Mullen DQ (p = 0.002). Combined groups, more intervention hours led to significant improvement in Mullen DQ (0.78, 95% CI 0.08 to 1.47) and Mullen Verbal DQ (1.09, 95% CI 0.11 to -2.06) (both p < 0.05) 2. Words and gestures (MCDI) (parent-reported): No significant group differences related to treatment. Combined groups, more intervention hours led to improvements on MCDI Vocab Comprehension (4.22, 95% CI 0.15 to 8.3) (p < 0.05) 3. Adaptive behaviour (VABS-II) (parent-reported): No significant group differences related to treatment 4. Autism symptoms (ADOS): Modified ADOS Social Affect scores decreased in both P-ESDM (d = -0.37) and community (d = -0.63) groups. Combined, more intervention hours led to significant improvement on ADOS Restrictive and Repetitive scores (-0.11, 95% CI -0.22 to 0, p < 0.05)	12 weeks diagnostic measure (ADOS-T): Both P-ESDM and community groups showed reduction in core autism symptoms, but 95% continued to meet criteria for ASD	No difference between P-ESDM and community treatment on parent outcomes 1. Use of interaction skills (ESDM Parent Fidelity Tool): Significant post-intervention gains in interaction skills in both P-ESDM group (d = 0.57, p = 0.001) and community group (d = 0.36, p = 0.029) 2. Working alliance with primary therapist: P-ESDM group reported significantly stronger working alliance with primary therapist than community intervention group (p = 0.06)
Kasari et al., (2014)	No significant treatment effects on child joint attention and language outcomes, although overall, children across both groups showed increases in Mullen language skills 1. Joint attention (ESCS): No significant changes in initiating and responding to joint attention between intervention and control groups 2. Language (Mullen): Increases in Visual Reception (p < 0.001), Expressive (p < 0.001) and Receptive (p < 0.001) Language scores in overall sample, with no significant differences related to intervention	1-year follow up (ADOS): Overall, 80% of children met criteria for ASD; 19/24 FPI and 20/25 controls, with no significant differences in joint attention and language outcomes. The 10 children without ASD were considered delayed, with greater improvement in expressive language (p = 0.04) compared to children with ASD	1.Responsiveness (percentage within 10-min play): FPI group showed significantly higher, improved parental responsiveness compared to control group (Cramer's V = 0.42, p = 0.001), this effect was not maintained at follow-up. Further analysis in parents who showed responsiveness at baseline (pre-treatment characteristic) found that those who underwent FPI had significantly increased responsiveness compared to controls (p = 0.02) and this effect was still significant at follow-up (p = 0.02)
Baranek et al., (2015)	1. Language (Mullen): Significant improvement in infants' Receptive Language as an effect of intervention (effect size* 0.876, p < 0.05); non-significant for Expressive Language 2. Communication (CSBS) (parent-reported): Significant improvement in Communication as an effect of intervention (effect size* 2.022, p < 0.05); non-significant for Behaviour 3. Sensory (SEQ) (parent-reported): Significantly higher Hyperresponsiveness reported by parents in intervention group (effect size* 1.441, p < 0.05), lowered Hyporesponsiveness (effect size* -1.187, p < 0.05) 4. Adaptive behaviour (VABS-II) (parent-reported): Higher VABS-II scores; Expressive Communication (effect size 0.701), Receptive Communication (effect size* 0.972) and Socialization (effect size* 1.968), all p < 0.05 *effect sizes regression-based analog of Cohen's D, results here were taken at Time 3	18–19 months follow up (ADOS and diagnostic interview): Overall, 44% of children obtained ASD diagnosis; 4/11 ART, 2/5 REIM and 2/2 Eligible/Declined diagnosed with ASD. The children without ASD were noted to have varying developmental concerns	1. Responsiveness (MBRS): No significant differences between intervention and control groups 2. Directiveness (MBRS): ART group showed significantly lower levels of directiveness (effect size -0.642, p < 0.05) at Time 3, treatment effects decreased between Time 2 (post-test) and Time 3
Green et al., (2015)	1. Infant attentiveness to parent (MACI): Slight intervention effect to improve infant attentiveness to caregiver, effect size 0.29 (95% CI -0.24 to 0.86), ranging from slight negative effect to large positive effect 2. Atypical behaviours (AOSI): Relatively large reduction on AOSI scores in intervention (4.15 point mean reduction) compared to controls (1.77 point mean reduction), effect size 0.50 (95% CI -0.15 to 1.08) 3. Attention disengagement (Gap-overlap task): Faster disengagement in intervention group, effect size 0.48 (95% CI -0.01 to 1.02), where difficulty disengaging is an early marker of ASD symptoms 4. Adaptive Behaviour (VABS-II) (parent-reported): Significant intervention effect on adaptive behaviour, p = 0.0005, with improved Socialization (effect size 0.42, 95% CI -0.07 to 0.98) but reduced Communication (effect size -0.36, 95% CI -1.04 to 0.31). At Follow-up 1 and 2, intervention effects were nonsignificant 5. Language (Auditory ERPs, Mullen and parent-reported MCDI vocabulary): No significant intervention effects	30-month follow up (ADOS-2) (Green et al., 2017): 4/27 (15%) VIPP, 2/26 (8%) controls diagnosed with ASD, with no intervention effect on diagnosis. 7/27 (26%) VIPP, 8/26 (31%) controls atypical development. The remaining children showed typical development	1. Sensitive-responding (MACI): No effect of VIPP on caregiver sensitive responding 2. Non-directiveness (MACI): Strong effect of VIPP in increasing caregiver non-directiveness (effect size 0.81, 95% 0.28 to 1.52), but this effect reduces by Follow-up 1 to extinction at Follow-up 2
Jones et al., (2017)	1. Speed of habituation to faces vs objects: PFR group showed significantly shorter habituation time to faces vs objects compared to A+M (p = 0.033, *η*^*2*^ = 0.17), effect carried forward at 12-month follow-up (p = 0.04, *η*^*2*^ = 0.19) 2. EEG theta power to social vs nonsocial stimuli: Significant increase in EEG-theta power at 6-month post-test to both social and nonsocial stimuli in PFR group vs A+M (p = 0.042, *η*^*2*^ = 0.18), this was not specific to social stimuli as hypothesized 3. ERP responses to faces/objects: At 6-month post-test, A+M infants showed larger P400 amplitude to objects than faces (p = 0.029, *η*^*2*^ = 0.65) than PFR infants. At 12-month follow-up, A+M infants showed less prolonged P400 responses to faces than objects (p = 0.025, *η*^*2*^ = 0.49) than PFR infants. Overall, PFR group's responses appear to closely match normative controls	Not reported	Not reported
Watson et al., (2017)	1. Social-Communication (CSBS) (parent-reported) & Sensory-Regulatory outcomes (SPA): No main effects of intervention 2. Development (Mullen): No main effects of intervention 3. Adaptive Behaviour (VABS-II) (parent-reported): No main effects of intervention overall, but authors found an effect of intervention on VABS Motor score (d = 0.65, 95% CI 2.32 to 11.69, p = 0.001) 4. Autism Symptoms (ADOS): No effects of intervention 5. Mediated by parent responsiveness (indirect treatment effect estimates in brackets): Decreased: SPA Hyperresponsiveness (-0.09), ADOS Total scores (-1.44). Increased: Mullen Expressive (2.54) and Receptive (3.32) Language, Mullen Fine Motor (2.45), VABS-II Communication (3.25) and Socialization (1.94) (all p < 0.05)	At post-intervention: 41% of sample across both groups met ADOS criteria for "Autism" and 30% met ADOS criteria for "Autism Spectrum"	1. Parent Responsiveness (PRCS): Intervention significantly increased parent responsiveness (d = 0.62, p < 0.05) 2. Responsiveness and Affect (MBRS): Intervention significantly increased responsiveness (d = 0.46, p < 0.05) and affect (d = 0.75, p < 0.01) 3. Parental Stress Scale: Initial lower burden and higher reward associated with Mullen Visual Reception and VABS Daily Living Skills
Whitehouse et al., (2019)	Overall, TAU group received significantly more community therapy (psychology and speech/language therapy) than the iBASIS-VIPP group (p < 0.0001) 1. Early ASD behavioural signs (AOSI): No significant treatment effects on AOSI scores 2. Infant positive affect (MACI): iBASIS-VIPP group had lower scores than TAU group (effect estimate -0.69, 95% CI -1.27 to -0.10). No significant treatment effects on MACI infant attentiveness 3. Motor and cognitive development (Mullen): No significant treatment effects on Mullen languages, visual reception and fine motor subscales 4. Communication subscale (VABS-II) (caregiver-reported): Positive treatment effect of iBASIS-VIPP (effect estimate 6.43, 95% CI 1.06 to 11.81) 5. Receptive & expressive language (MCDI) (caregiver-reported): Positive treatment effect of iBASIS-VIPP on receptive (effect estimate 37.17, 95% CI 10.59 to 63.75) and expressive language (effect estimate 2.31, 95% CI 1.22 to 4.33)	6 months post intervention (AOSI): No statistically significant treatment effects on early ASD symptoms	1. Caregiver sensitive responding and non-directiveness (MACI): No significant treatment effects 2. Parenting Sense of Competence (PSOC) (caregiver-rated): No significant treatment effects on any subscales

*ADOS* Autism Diagnostic Observational Scale, *ADOS-T* Autism Diagnostic Observational Scale Toddler, *A*+*M* Assessment and Monitoring, *AOSI* Autism Observation Scale for Infants, *ART* Adapted Responsive Teaching, *ASD* Autism Spectrum Disorder, *CI* Confidence Interval, *CSBS* Communication and Symbolic Behaviour Scales, *DQ* Developmental Quotient, *EEG* Electroencephalogram, *ERP* Event-Related Potential, *ESCS* Early Social Communication Scale, *FPI* Focused Playtime Intervention, *iBASIS-VIPP* British Autism Study of Infant Siblings-Video Interaction for Promoting Positive Parenting, *MACI* Manchester Assessment of Caregiver–Infant Interaction, *MBRS* Maternal Behaviour Rating Scale, *MCDI* MacArthur-Bates Communicative Development Inventory, *Mullen* Mullen Scales of Early Learning, *P-ESDM* Parent-Delivered Early Start Denver Model, *PFR* Promoting First Relationships, *PRCS* Parent Responsiveness Coding System, *REIM* Referral to EI and Monitoring, *SEQ* Sensory Experiences Questionnaire, *SPA* Sensory Processing Assessment, *TAU* Treatment as usual, *VABS-II* Vineland Adaptive Behaviour Scales, Second Edition

### Participants: Sampling Methods and Determinants of Autism Risk

Across seven studies, there were 457 participants with a mean age under 24 months, with age range spanning 6 to 31 months. ASD risk status were defined either by familial risk or by early ASD screening. Two studies (n = 87) conducted on infants under 12 months did not screen infants for atypical neurodevelopment (Green et al., 2015; Jones et al., 2017). In these studies, the determinant of ASD risk was having an older sibling with ASD. Green et al. (2015) recruited participants from the British Autism Study of Infant Siblings (BASIS), a large-scale research network of infant siblings of children with ASD in the UK. In their supplementary materials, Jones et al. (2017) described recruiting high-risk infant siblings after administering the Autism Diagnostic Interview-Revised (ADI-R) on their older brother or sister with ASD. The latter study did not report sampling location or recruitment methods.

Five studies (n = 370), with infants between 12 to 31-months-old, used standardized instruments to determine participants’ ASD risk. Two studies screened infants from community samples using the First Year Inventory (FYI) (Baranek et al., 2015; Watson et al., 2017). In addition to the FYI, Baranek et al. (2015)’s eligibility procedures also included pre-intervention instruments (see Table 2) to ensure that infants met ASD diagnostic cut-offs, showed delays in social-communication skills and had disruptions in sensory-regulatory functions. The following three studies recruited infants from paediatricians, autism clinics, research programmes and government services. Rogers et al. (2012) used two screening instruments; Infant Toddler Checklist (ITC) for all infants, Early Screening of Autistic Traits Questionnaire (ESAT) for infants between 12 to 15 months and Modified Checklist for Autism in Toddlers (M-CHAT) for infants between 16 to 24 months. Kasari et al. (2014) recruited infants who met concerns on the M-CHAT and Communication and Symbolic Behaviour Scales Developmental Profile (CSBS DP). Lastly, Whitehouse et al. (2019) screened infants who were accessing government services or referred by community health nurses using the Social Attention and Communication Surveillance-Revised (SACS-R) checklist.

### Intervention Characteristics

#### Types of Interventions

There were five distinct types of interventions across seven studies reviewed; Parent-delivered Early Start Denver Model (P-ESDM) (Rogers et al., 2012), Focused Playtime Intervention (FPI) (Kasari et al., 2014), Adapted Responsive Teaching (ART) (Baranek et al., 2015; Watson et al., 2017), Video Interaction for Promoting Positive Parenting adapted for British Autism Study of Infant Siblings (iBASIS-VIPP) (Green et al., 2015; Whitehouse et al., 2019), and Promoting First Relationships (PFR) (Jones et al., 2017). The mechanism of change in all interventions were aimed at developing reciprocal social communication skills between parents and children. All interventions were adaptations of existing interventions, with modifications made to target ASD in infants.

P-ESDM (Rogers et al., 2012) was adapted from the therapist-delivered Early Start Denver Model (ESDM), an intervention promoting social and emotional development in very young children with ASD using Applied Behavioural Analysis techniques (Vismara et al., 2009). ART was adapted from Responsive Teaching, an early intervention approach for caregiver and professionals working with children with developmental disabilities. Modifications were made to ensure that ART was suitable for infants at risk for ASD in two studies (Baranek et al., 2015; Watson et al., 2017). Based on positive parenting interventions, FPI, iBASIS-VIPP and PFR were also adapted to target atypical behaviours in ASD. FPI focused on developing family-centred play techniques to enhance parents’ responsivity and establish parent–child balance in play (Kasari et al., 2014). iBASIS-VIPP was adapted from the original Video Interaction to Promote Positive Parenting (VIPP) programme to address atypical communicative behaviours in infants at risk for ASD. This was used in the UK and Australian studies (Green et al., 2015; Whitehouse et al., 2019). PFR was developed to facilitate interactions between parents and infants who present with a wide range of developmental risk factors using positive parenting techniques (Jones et al., 2017).

#### Lengths and Intensities of Interventions

Duration of interventions ranged from 10 weeks (PFR) to 34 weeks (ART). In terms of intensity, almost all interventions consisted of 60- to 90-min sessions conducted weekly. The ART interventions had gradually decreasing intensities, such that these started with two home sessions weekly for the first 6 weeks, which was reduced to one home session and one phone call weekly, which was further reduced to one home session weekly. The iBASIS-VIPP interventions lasted up to 2 h per session, but these were conducted either weekly or fortnightly depending on family needs.

#### Locations of Interventions and Assessments

Four interventions, FPI, ART, iBASIS-VIPP and PFR, conducted parent-training in the infants’ homes (Baranek et al., 2015; Green et al., 2015; Jones et al., 2017; Kasari et al., 2014; Watson et al., 2017; Whitehouse et al., 2019). The P-ESDM parent-training were conducted at university clinics (Rogers et al., 2012). All studies conducted outcome assessments at university laboratories or community centres. The PFR study did not explicitly report the location of assessments (Jones et al., 2017), but due to the nature of eye-tracking and electroencephalography tasks, this review considers them to be conducted in laboratories.

#### Therapist Fidelity

All interventions were delivered by trained interventionists or therapists, and therapist fidelity was reported in all studies. In the two ART studies, fidelity was measured on the Implementation Fidelity Checklist (IFC). One study (Baranek et al., 2015) reported fidelity scores ranging from 80 to 90% across sessions, while the second study (Watson et al., 2017) reported the mean IFC proportional score of 0.87 (within the “good” range). Therapist fidelity score in the P-ESDM was an average of 3.62 (range of 1 to 4) (Rogers et al., 2012). In the iBASIS-VIPP interventions, both studies passed the 80% fidelity scores; mean fidelity score was 19.4 (range of 15 to 21) in the UK study (Green et al., 2015) and 20.5 (range of 18 to 21) in the Australian study (Whitehouse et al., 2019). In the FPI study, the average fidelity was at 94% (Kasari et al., 2014). Fidelity coding was described in the supplementary materials of the PFR study (Jones et al., 2017), where the sole PFR provider passed 100% of fidelity checks.

### Risk of Bias

#### Random Sequence Generation; Allocation Concealment

Considering all studies included in the review were RCTs, randomization and blinding should be central to the procedures. Percentage agreement on risk of bias ratings was 100% for all seven studies in these two domains (κ = indeterminate for randomization; κ = 1.00 for allocation concealment). Indeed, all studies described randomization sequences and blocks used in their studies, and stratification processes were also described if used. Six studies (Baranek et al., 2015; Green et al., 2015; Kasari et al., 2014; Rogers et al., 2012; Watson et al., 2017; Whitehouse et al., 2019) reported using independent statisticians, data coordinating centres and data management staff to conduct these randomization procedures. However, one study did not report this information (Jones et al., 2017). As such, all studies had low risk of bias for random sequence generation and allocation concealment, except for unclear risk of bias in allocation concealment in one study (Jones et al., 2017).

#### Blinding of Participants and Personnel

Due to the nature of parent-mediated interventions, it was not possible to blind parents and therapists delivering the training. Additionally, most studies used treatment-as-usual or community intervention as control groups, which would have naturally revealed families’ assignments throughout the study period. Agreement between raters was 57.1% (κ = -0.17, 95% CI: -0.41, 0.08), with moderate disagreement on two studies (Jones et al., 2017; Kasari et al., 2014) and substantial disagreement on one study (Green et al., 2015). Disagreements were resolved by adhering to the Cochrane criteria (Higgins et al., 2011) and exercising objectivity in the judgement of evidence reported. As a result, six studies were rated with high risk of bias for unlikely blinding of participants and personnel (Baranek et al., 2015; Green et al., 2015; Jones et al., 2017; Rogers et al., 2012; Watson et al., 2017; Whitehouse et al., 2019). Only one study (Kasari et al., 2014) used an “active” control group which involved four 90-min home sessions throughout the 12-week programme, where interventionists delivered strategies to help parents address challenging behaviours and enhance social-emotional competence. However, this study had an “unclear” risk of bias as it was not specified if parents and interventionists were blind to group assignment.

#### Blinding of Outcome Assessment

Different combinations of caregiver- and personnel-reported measures were used to assess outcomes across the studies reviewed. There is a high likelihood of performance bias among parents and caregivers who received interventions, especially in rating their own infants at post-test. Agreement between raters was lowest in this domain, with 28.6% (κ = indeterminate) agreement on two studies rated with low risk of bias, as they solely used blinded personnel-rated outcome assessments (Jones et al., 2017; Kasari et al., 2014). There was moderate disagreement (low vs unclear) on the remaining five studies, which on final consensus were rated with “unclear” risk of bias, as combinations of blinded personnel-reported measures and non-blinded parent-reported measures were used, and it is difficult to determine if this would have affected the concluding outcomes. Among these, four studies (Baranek et al., 2015; Green et al., 2015; Watson et al., 2017; Whitehouse et al., 2019) reported using blind assessors to rate personnel-reported measures while one study (Rogers et al., 2012) did not specify if assessors were blind to group assignment.

#### Incomplete Outcome Data

Due to the level of commitment and time spent in interventional studies, participant attrition is anticipated. Percentage agreement was 100% (κ = 1.00) for all seven studies in this domain. Five studies were rated with low risk of bias as attrition rates were reported and suitably addressed in statistical analyses (Baranek et al., 2015; Green et al., 2015; Kasari et al., 2014; Watson et al., 2017; Whitehouse et al., 2019). One study had unclear risk of bias as dropouts were not reported and incomplete data in results were not addressed by the authors (Rogers et al., 2012). Finally, one study had high risk of bias as data collection depended on infants’ compliance with tasks such as EEG (Jones et al., 2017), leading to large variance in dropouts across different measures taken by the researchers.

#### Selective Reporting

In this criterion, we sought evidence of protocols, pre-planned analyses or description of statistical analyses in the studies. Percentage agreement between raters was 100% (κ = 1.00) for all seven studies. Two studies met low risk of bias for registering study protocols and sufficiently explaining the changes made before analyses were undertaken (Watson et al., 2017; Whitehouse et al., 2019). Two other studies also met low risk of bias for reporting pre-planned analyses or sufficient statistical analyses prior to reporting results (Green et al., 2015; Kasari et al., 2014). Two studies had unclear risk of bias; one due to partially explained analytic approaches (Rogers et al., 2012) and another due to reporting of effect sizes based on statistical significance only (Baranek et al., 2015). Lastly, one study described conducting pre-planned analysis but was rated with high risk of bias due to insufficient reporting of data and results (Jones et al., 2017).

#### Other Bias—Reporting of Effect Sizes

Here, we review the description of effect sizes in the studies. Percentage agreement was high at 85.7% (κ = 0.68, 95% CI: 0.27, 1.00), with moderate disagreement on one study (Jones et al., 2017). Five studies were rated with low risk of bias as they reported the measurements of effect sizes. Of these, two studies reported Cohen’s d measures (Rogers et al., 2012; Watson et al., 2017), with variations in between- and within- group calculations. One study reported Cohen’s d and Cramer’s V (Kasari et al., 2014). Two other studies reported effect estimates generated from group differences at endpoint using regression analyses (Green et al., 2015) and ANCOVA regression models (Whitehouse et al., 2019). Two studies had unclear risk of bias. Of these, one study described using a regression-based analog of Cohen’s d but did not report full results of effect sizes (Baranek et al., 2015), while another reported eta-squared (η^2^) measures but it is unclear if the published results were complete (Jones et al., 2017).

#### Other Bias: Disclosure of Conflict of Interests

Crucially, studies should fully disclose conflicts of interests due to the potential for these factors to influence the research process (Bekelman et al., 2003; Cortese et al., 2016). Disclosures may not necessarily lead to high risk of bias in studies (Singh et al., 2020), but it is important to assess disclosures collectively with the other Cochrane domains. Percentage agreement was 100% (κ = 1.00) for all seven studies in this domain. Out of seven studies reviewed, only one study did not provide a statement on conflict of interests (Kasari et al., 2014). For this reason, this study was rated with unclear risk of bias. Three studies disclosed conflicts of interests and were rated high; including authors receiving royalties from intervention materials (Rogers et al., 2012), book authorship royalties and positions in research and pharmacological companies (Jones et al., 2017), and authors declaring intellectual property rights to instruments used in the study (Watson et al., 2017). Three studies declared no competing interests and were rated with low risk of bias (Baranek et al., 2015; Green et al., 2015; Whitehouse et al., 2019).

#### Summary

**Fig. 2:**
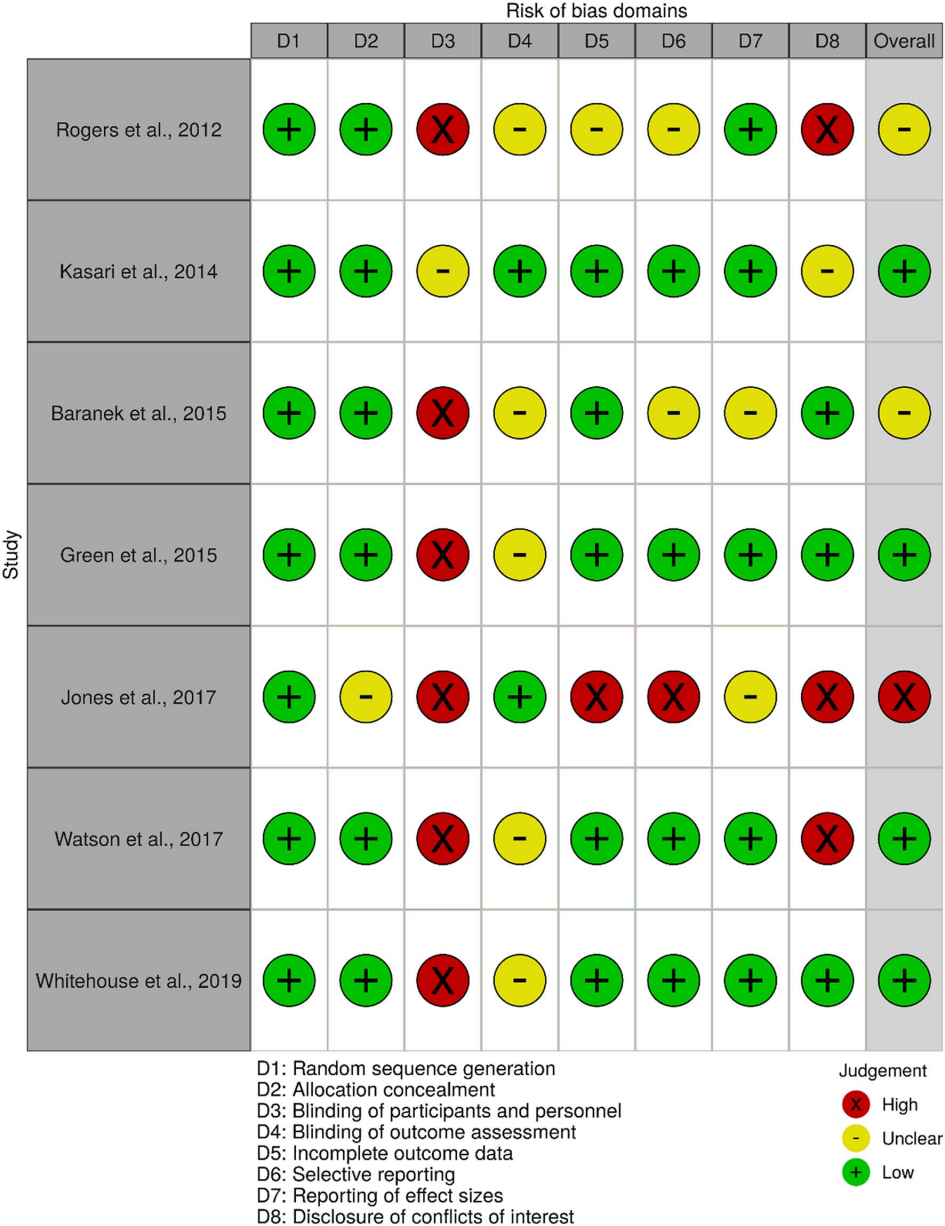
Assessment of risk of bias in studies based on Cochrane Collaboration’s tool (Higgins et al., 2011), with items D7 and D8 added for the purposes of this review. Risk of bias plot was created using *robvis* (McGuinness & Higgins, 2020)

The risk of bias assessment is summarized in Fig. 2. The overall judgements were made in consideration of the nature of interventional research in this review; in particular, the challenges of blinding parents from assignment groups and outcome assessments. Percentage agreement on the overall judgements was high at 85.7% (κ = 0.73, 95% CI: 0.25, 1.00), with moderate disagreement on one study (Baranek et al., 2015). Overall, four studies met criteria for low risk of bias (Green et al., 2015; Kasari et al., 2014; Watson et al., 2017; Whitehouse et al., 2019), two studies had unclear risk of bias due to multiple concerns which cannot be clarified from the articles (Baranek et al., 2015; Rogers et al., 2012), and one study had high risk of bias (Jones et al., 2017).

### Outcomes Assessment

Outcomes of studies were extracted and summarized in Table 3. Outcomes were assessed pre- and post-intervention, with four studies (Baranek et al., 2015; Green et al., 2017; Jones et al., 2017; Kasari et al., 2014) reporting follow-up data ranging from 12 to 30 months after the baseline. One study (Whitehouse et al., 2019) reported planned follow-ups at 12 months and 18 months after baseline but these results are not yet available. In this section, infant outcomes, parent outcomes and mediating factors will be described. Outcomes rated by parents or caregivers are indicated accordingly. These results should be viewed in consideration of the risk of bias assessments described above, especially variations of ASD risk in samples, use of blinded outcome assessments and measurements of effect sizes in the studies.

#### Infant Outcomes

A variety of infant outcomes were assessed on up to ten caregiver- and researcher-reported measures across studies. Two studies also used eye-tracking and electroencephalography (EEG) tasks (Green et al., 2015; Jones et al., 2017). In this review, the measures used are broadly categorized according to the outcomes being assessed, such as diagnostic, developmental, adaptive behaviour, communication, social attentional skills and sensory measures. In the studies reviewed, ASD diagnostic measures included the Autism Observation Scale for Infants (AOSI, n = 3) and the Autism Diagnostic Observation Scale-Toddler (ADOS-T, n = 5). Developmental outcomes were measured on Mullen Scales of Early Learning (MSEL, n = 6) while adaptive behaviours were assessed on Vineland Adaptive Behaviour Scales (VABS-II, n = 5). Communication skills, including language, were measured on the MacArthur-Bates Communicative Developmental Inventory (MCDI, n = 4) and Communication and Symbolic Behaviour Scales (CSBS, n = 2). Social attentional skills were measured on Manchester Assessment of Caregiver-Infant Interaction (MACI, n = 2), eye-tracking tasks (n = 2), attention orientation tasks (n = 1) and Early Social Communication Scale for joint attention (ESCS, n = 1). Lastly, the Sensory Experiences Questionnaire (SEQ, n = 2) and Sensory Processing Assessment (SPA, n = 2) were used to measure sensory sensitivities.

#### ASD Diagnosis

Diagnostic outcomes were reported in six studies (Baranek et al., 2015; Green et al., 2015; Kasari et al., 2014; Rogers et al., 2012; Watson et al., 2017; Whitehouse et al., 2019). None of the studies found evidence for significant intervention effects on reducing risk of ASD diagnosis. In their follow-up reports, Green et al. (2017) observed significant treatment effects on reduction of autism prodromal symptoms on the AOSI and ADOS-2 up to 24 months after intervention. Another study (Rogers et al., 2012) reported a reduction in core autism symptoms (ADOS social affect scores) in both intervention and control groups, but 95% of their participants still met criteria for ASD post-intervention. However, their findings also indicated that overall increased intervention (P-ESDM and community) hours led to improved ADOS Restrictive and Repetitive scores. Watson et al. reported mediation effects; where increased parental responsiveness was significantly associated with decreased ADOS scores (p < 0.05) (Watson et al., 2017). However, there were no direct intervention effects on autism symptoms. Additionally, infants who did not meet criteria for ASD were reported to have developmental concerns in three studies (Baranek et al., 2015; Green et al., 2015; Kasari et al., 2014). In short, none of the interventions directly influenced ASD diagnosis in at-risk infants.

#### Mullen Scales

Developmental outcomes were assessed on the Mullen scales (MSEL) in six studies. Mixed results were reported for ART; while one study (Baranek et al., 2015) found that infants in the ART group showed statistically significant increases in Mullen receptive language (effect size 0.876, p < 0.05), the later study (Watson et al., 2017) found no main effects of ART on any developmental outcomes. P-ESDM and FPI had no treatment effects on the Mullen scores, as all infants showed significant improvements at post-test regardless of treatment groups. In the P-ESDM study (Rogers et al., 2012), significant improvements on the Mullen overall scores were significantly associated with younger child age (p = 0.002) and increased intervention hours (p ≤ 0.05), not intervention group. In the FPI study, infants’ scores were significantly increased across the Mullen scales for the overall sample (p < 0.001) (Kasari et al., 2014). Meanwhile, both iBASIS-VIPP studies reported no significant intervention effects on the Mullen scales (Green et al., 2015; Whitehouse et al., 2019). In short, where significant improvements were present on the Mullen scales, these were present across intervention and control groups. Although significant effects were reported in one ART intervention, this result was not replicated in the later study (Baranek et al., 2015; Watson et al., 2017).

#### VABS-II

Adaptive behaviour outcomes were measured on the parent-reported VABS-II in five studies. P-ESDM had no significant treatment effect on infants’ adaptive behaviour (Rogers et al., 2012). ART interventions reported mixed results. Baranek et al. (2015) reported increased VABS-II scores in the intervention group, consisting of Expressive Communication, effect size 0.701; Receptive Communication, effect size 0.972; Socialization, effect size 1.968; all p < 0.05). The later study by Watson et al. found a single treatment effect on VABS-II Motor scores (d = 0.65, p = 0.001), although the authors highlighted that this result may reflect a regression to the mean. Further analysis found that treatment effects on VABS-II Communication and Socialization scores (p < 0.05) were mediated by higher parental responsiveness (Watson et al., 2017). The iBASIS-VIPP interventions produced mixed results. In Green et al.’s study, there were significant intervention effects on overall adaptive behaviour (p = 0.0005); this consisted of improved Socialisation (effect size 0.42) with reduced Communication (effect size -0.36) (Green et al., 2015), but the results were no longer significant at 30-month follow-up. In contrast, Whitehouse et al. reported positive treatment effects on the Communication subscale only (effect size 6.43) (Whitehouse et al., 2019). Overall, there appears to be mixed evidence for intervention effects on communication and socialisation subscales on the VABS-II (Baranek et al., 2015; Green et al., 2015; Whitehouse et al., 2019), which were mediated by parental interaction in one study (Watson et al., 2017).

#### Social Attentional Skills

Multiple measures were used across studies to measure social attentional skills, limiting the comparability of the reported results. P-ESDM (Rogers et al., 2012) and FPI (Kasari et al., 2014) had no statistically significant intervention effects on mean percentages of joint attention as measured on eye-tracking tasks and the ESCS, respectively. In Green et al. (2015)’s study, iBASIS-VIPP had a moderate effect size on increasing infant attentiveness to parent (measured on the MACI), but this ranged from a slight negative effect to a large positive effect of treatment (effect size 0.29, 95% CI -0.24 to 0.86). The authors also reported significantly improved attention disengagement (effect size 0.48, 95% CI -0.01 to 1.02) using eye-tracking tasks, indicating improvement to an early marker of ASD risk. These results were not replicated in Whitehouse et al. (2019)’s iBASIS-VIPP study; there were no effects of intervention on the MACI infant attentiveness subscale. In the PFR study (Jones et al., 2017), attentional engagement was measured using EEG, and it was hypothesized that intervention would lead to increased theta responses when infants are viewing social (vs non-social) stimuli. While infants in the intervention group indeed showed increased EEG-theta power compared to the control group (p = 0.042), this was in response to both social and non-social stimuli. Overall, these results drew indefinite conclusions.

#### Communication

In the studies reviewed, infants’ communication skills were measured on two caregiver-reported measures, MCDI (four studies) and CSBS (two studies). No significant treatment effects were found on the MCDI in three studies, namely the P-ESDM (Rogers et al., 2012), ART (Watson et al., 2017) and iBASIS-VIPP (Green et al., 2015) interventions. Whitehouse et al.’s iBASIS-VIPP study (Whitehouse et al., 2019) found positive treatment effects on MCDI Receptive Language (effect size 37.17, 95% CI 10.59 to 63.75) and Expressive Language (effect size 2.31, 95% CI 1.22 to 4.33). The CSBS measure was used in the two ART interventions. Baranek et al. (2015) reported a significant (p < 0.05) improvement in CSBS Communication as an effect of intervention (effect size 2.022), but these results were not replicated by the later study (Watson et al., 2017) which found no effects of intervention on any of the scales. In short, while significant improvements had been reported on MCDI (Whitehouse et al., 2019) and CSBS (Baranek et al., 2015) subscales, these were not replicated in studies using the same interventions.

#### Sensory Sensitivities

Responses to sensory stimuli were assessed in the two ART intervention studies. The Sensory Experiences Questionnaire (SEQ) is a parent-report measure while the Sensory Processing Assessment for young children (SPA) is rated by trained assessors. In the first study (Baranek et al., 2015), parents in the ART intervention reported significantly higher SEQ Hyperresponsiveness (effect size 1.441, p < 0.05) but lower SEQ Hyporesponsiveness (effect size -1.187, p < 0.05) in infants. No intervention effects were found on the SPA scales. In the second study by Watson et al. (2017), no direct intervention effects were found on both the SEQ and SPA. However, treatment effects were mediated by parent responsiveness on lowered SPA Hyperresponsiveness. Results between parent-report and researcher-report measures appear to be inconsistent in the two studies.

#### Parent Outcomes

In this section, we will describe parental outcomes. Four studies assessed parental outcomes using validated measures (Baranek et al., 2015; Green et al., 2015; Watson et al., 2017; Whitehouse et al., 2019). These measures include the Maternal Behaviour Rating Scale (MBRS) (used in both ART intervention studies), Manchester Assessment of Caregiver-Infant Interaction (MACI) subscales (used in both iBASIS-VIPP studies) and caregiver-rated Parenting Sense of Competence (PSOC) (used in Whitehouse et al.’s iBASIS-VIPP study). Three studies used non-validated measures (including behavioural coding) (Kasari et al., 2014; Rogers et al., 2012; Watson et al., 2017). These are the ESDM Parent Fidelity Tool (used in the P-ESDM study), percentage scores based on coding of parental behaviour (in the FPI study), and the Parent Responsiveness Coding System (PRCS) (used in Watson et al.’s ART study). Finally, one study did not assess parental outcomes (Jones et al., 2017).

#### Validated Measures: MBRS, MACI, PSOC and Parental Stress Scale

The MBRS measures parental interactions across four dimensions: responsive, affect, directive and achievement orientation. Baranek et al. (2015) assessed the effects of ART on two of these outcomes (responsive and directive), and found significant intervention effects in lowering parental directiveness (effect size -0.642, p < 0.05) at post-test. Watson et al. (2017) assessed the effects of ART on all four dimensions, and reported that intervention significantly increased parental responsiveness (d = 0.46, p < 0.05) and affect (d = 0.75, p < 0.01). Parent outcomes on the MACI include caregiver sensitive responsiveness and non-directiveness. Green et al. (2015) found a strong effect of iBASIS-VIPP on increasing caregiver non-directiveness (effect size 0.81) at post-test, but this effect was not retained at follow-up. Using the same intervention, Whitehouse et al. (2019), who used both the MACI and caregiver-rated PSOC scales, found no effects on any parent outcomes. Overall, a number of positive intervention effects were reported, although these results were not replicated in separate studies on the same interventions, namely ART (Baranek et al., 2015; Watson et al., 2017) and iBASIS-VIPP (Green et al., 2015; Whitehouse et al., 2019).

#### Non-Validated Measures

On the ESDM Parent Fidelity Tool, interaction skills were defined as child-centred, responsive interactive styles displayed by parents (Rogers et al., 2012). Rogers et al. found significant gains in interaction skills in all parents (P-ESDM, effect size 0.57, p = 0.001; control, effect size 0.36, p = 0.029), with no significant differences between intervention and control groups. In the FPI intervention, parents’ acts of responding, directing or ignoring play acts were blind-coded and converted into percentage scores (Kasari et al., 2014). The primary outcome was measured by the proportion of times the parent was responsive, where the FPI group showed significantly improved percentage responsiveness compared to the control group (Cramer’s V = 0.42, p = 0.001) at post-test, but not at follow-up. In the second ART study, PRCS was used to measure percentage of parental responses to children (Watson et al., 2017). Their results showed that intervention led to significantly increased parent responsiveness (d = 0.62, p < 0.05). Taken together, studies report positive gains on parental outcomes on both validated and non-validated measures.

#### Moderating Factors

The validated, self-reported Parental Stress Scale was used to measure initial parent Burden and Reward factors in one ART study (Watson et al., 2017). These factors were found to be moderators of treatment effects. In the ART group, infants of parents with higher Reward and lower Burden showed significant improvement on Mullen Visual Reception (p = 0.045). Initial parenting Reward was also positively correlated with VABS Daily Living Skills. There appears to be an impact of assignment group on the moderation effects of these factors, as reverse patterns were reported in the control groups although these were non-significant. The authors noted that these results are preliminary due to small sample sizes.

#### Mediating Factors

Only one study in this review (the second ART intervention study) reported statistically significant mediating factors. Watson et al. (2017) reported that ART significantly increased parent responsiveness (p < 0.05) on both MBRS (d = 0.46) and PRCS (d = 0.62) measures, which mediated intervention effects on a number of outcomes. These included decreases in SPA Hyperresponsiveness (effect estimates -0.09) and ADOS total scores (-1.44), increases in Mullen Expressive Language (2.54), Receptive Language (3.32) and Fine Motor (2.45) subscales and increases in VABS-II Communication (3.25) and Socialisation (1.94), all of which were deemed statistically significant at p < 0.05. The FPI study (Kasari et al., 2014) assessed mediating effects of percentage parental responsiveness and number of intervention hours on Mullen Developmental Quotient, Expressive and Receptive Language and ESCS Joint Attention, but results did not show statistically significant associations. These results are insufficient to draw the necessary inferences.

## Discussion

The objective of this review was to critically evaluate the potential of parent-mediated interventions in the first two years of life in infants at risk for ASD. Seven RCTs with a diverse range of interventions were identified, illustrating the heterogeneity of interventional research in this field. Based on the current available evidence in this review, interventions appear to have no direct impact on autism symptoms or influenced the risk of eventual ASD diagnoses. Overall, inconsistent results were found for post-intervention effects on infant outcomes across various domains including developmental, adaptive behaviour, communication, social attentional skills and sensory sensitivities. However, the studies provided moderate evidence for positive intervention effects on parents’ interaction styles on validated and non-validated measures. Positive mediation effects of improved parental interaction leading to improved infant outcomes were reported in one study (Watson et al., 2017). Studies that used the same interventions did not replicate results. However, definitive conclusions about the efficacy of these interventions are unfeasible at this point because of substantial heterogeneity, use of different outcome measures and varying effect size calculations.

The RCTs reviewed were comparing the effects of parent-based interventions against control groups, most of whom were receiving community care. Five out of seven studies reported that some improved infant and parent outcomes were seen in both groups (Baranek et al., 2015; Kasari et al., 2014; Rogers et al., 2012; Watson et al., 2017; Whitehouse et al., 2019). In all studies reviewed, a majority of families in control groups were referred to, or already accessing, community services. However, we do not have sufficient information about the type or intensity of intervention received in the control groups. Control groups may be motivated to seek external interventions in response to early screening results and randomization assignment (Rogers et al., 2012). As such, the absence of significant treatment effects may not indicate that the interventions were ineffective. Parent-mediated interventions may not be superior to interventions offered by community services (Baranek et al., 2015; Whitehouse et al., 2019), but they can possibly precede or supplement intensive interventions delivered by experts (Kasari et al., 2014; Webb et al., 2014). For precise comparisons, it is important for future studies to collect sufficient information on external interventions accessed by both treatment and control groups.

The current evidence provides groundwork for future research to build upon. It is essential to address limitations in these studies, especially the heterogeneity of recruited samples, measurement of outcomes and analytical approaches. Firstly, different recruitment methods, determinants of ASD risk and eligibility criteria were used across studies. Participants were recruited through community screening (Baranek et al., 2015; Watson et al., 2017), community services’ referrals (including autism clinics) (Kasari et al., 2014; Rogers et al., 2012; Whitehouse et al., 2019) and participation in research networks (Green et al., 2015) which may reflect different levels of parental motivations. These differences were augmented by differences in the determinants of ASD risk and eligibility criteria. Across studies, the inclusion criteria consisted of infant siblings with familial risk of ASD (Green et al., 2015; Jones et al., 2017), infants who met cut-offs on early screening measures such as the FYI (Watson et al., 2017; Whitehouse et al., 2019), and infants who clearly displayed atypical behaviours based on ASD-specific assessments (Baranek et al., 2015; Kasari et al., 2014; Rogers et al., 2012). There is an urgent need to clarify “ASD risk”, which should be addressed by future systematic reviews in this specific area (Levante et al., 2019).

Studies also differed considerably in measurement of outcomes and analytical approaches, as highlighted by earlier reviews in this area (Bradshaw et al., 2015; French & Kennedy, 2018; Oono et al., 2013). This leads to multiple challenges. Firstly, lack of consensus on outcome measures limits comparability between studies. Secondly, this raises concerns about the necessity of administering multiple instruments and increased chances of finding statistically significant results. Five of the studies reviewed administered different combinations of caregiver- and researcher-reported assessments, resulting in a range of six to 10 instruments used per study, including variations in multiple subscales within each instrument (Baranek et al., 2015; Green et al., 2015; Rogers et al., 2012; Watson et al., 2017; Whitehouse et al., 2019). In one study, authors declared intellectual property rights to three of the 10 instruments used, which may have influenced their study design (Watson et al., 2017). In contrast, another study (Kasari et al., 2014) used only three researcher-reported assessments, including the Mullen scales and ESCS, suggesting that fewer instruments might sufficiently measure outcomes. One study (Jones et al., 2017) used neurocognitive measures (i.e., EEG), removing biases associated with observational measures. However, these results should be interpreted with caution, pending further research into the role of EEG in identifying early biomarkers in ASD and effects of brain maturation on neurocognitive measures (Gabard-Durnam et al., 2019). In short, future studies should strongly consider standardizing the use of essential caregiver- and blinded researcher-reported assessments. Despite apparent parent bias in studies (Baranek et al., 2015; Whitehouse et al., 2019), information from caregivers remain essential to supplement researcher-rated measures.

Different analytic approaches were observed across studies, notably in the calculation of effect sizes. Researchers are generally encouraged to report effect sizes in quantitative studies as statistical significance or *p*-values are deemed inadequate to illustrate the sizes of differences between groups (Bakker et al., 2019; Maher et al., 2013). The challenges in comparing effect sizes are recognized (Smith et al., 2016) due to the variety of effect size calculations and other corresponding factors leading to increased heterogeneity in studies (Bakker et al., 2019). Within the present review, the effect sizes reported included Cohen’s *d*, Cramer’s *V*, eta-squared and effect estimates generated from the main analyses. These variations extend to the comparisons of effect sizes based on the calculation of within-group (Green et al., 2015; Rogers et al., 2012) or between-group differences (Kasari et al., 2014; Watson et al., 2017; Whitehouse et al., 2019). It will be important to have access to patient-level outcomes data in order to conduct analyses in the future to address this issue and provide better comparability of results across studies.

Given the fundamental role played by parents in these interventions, there also appears to be inadequate evaluation of parental factors. The preliminary results in one study (Watson et al., 2017) showed that initial parental stress and group assignment have significant impact on infant outcomes. Apart from that, all but one study (Jones et al., 2017) reported demographic information such as age, ethnicity and educational levels, and only Green et al. (2015) had information on maternal mental health or physical disorders. Parental age and positive parenting styles have been associated with outcomes in children with developmental disabilities including autism (Dyches et al., 2012). Naturally, parents with infants at risk for ASD would experience negative emotions associated with the burden of their children’s additional needs (Freuler et al., 2014), and parenting stress can also interact with ASD screening scores to later effect developmental competence (Nguyen et al., 2019). With increased knowledge of the familial risk of ASD, parents of infant siblings have reported a range of negative emotions associated with the uncertainties in early years (MacDuffie et al., 2020). Parental mental health is an important consideration in future studies, on top of parenting styles (Dyches et al., 2012), engagement (Haine-Schlagel et al., 2020) and stress levels (Strauss et al., 2012).

### Limitations of the Present Review

The present review identified a small number of RCTs, where four studies had low risk of bias, two studies had unclear risk of bias and one study had high risk of bias based on the Cochrane Collaboration’s tool (Higgins et al., 2011). This review recognizes that the subjective risk of bias assessments may not accurately reflect the quality of RCTs involved. Due to the nature of parent-delivered interventions, all studies met high or unclear risk of bias in blinding of parents and intervention personnel. Blinding of parents can potentially be achieved with active control groups (Kasari et al., 2014), but this raises ethical concerns. Families may be less likely to engage in community services if they believed their child’s needs are being met in research studies (Baranek et al., 2015). Considering the urgency of initiating early interventions for autism, blinding of parents may not be warranted if outcomes were rated by both parents and blinded assessors (Oono et al., 2013).

Overall, the studies identified for this review had small sample sizes, ranging from 16 to 103 infants per study. Earlier reviews on parent- and therapist-delivered interventions for ASD in children up to 6 years of age had reported similar issues with sample sizes (Bradshaw et al., 2015; McConachie & Diggle, 2007). Based on the studies reviewed, up to 30% of parents declined participation prior to interventions, even after their infants had passed eligibility tests (Jones et al., 2017; Kasari et al., 2014; Rogers et al., 2012; Watson et al., 2017; Whitehouse et al., 2019). Motivation to participate will likely differ between parents recruited from specialist clinics or community samples, which needs to be addressed in future studies (Watson et al., 2017). As described in the previous section, parental stress, psychopathology and coping styles could be significant elements in studies, along with other factors affecting participation. Further understanding of the needs and motivations of families may contribute to improved engagement in community or research interventions in the long-term (Haine-Schlagel et al., 2020; Pickard et al., 2017), especially those facing uncertainties with their infants’ “at-risk” status.

The conclusions in this review identifies the numerous methodological limitations in studies, such as differing recruitment methods, determinants of ASD risk, eligibility criteria, outcome measures and reporting of effect sizes. Increased heterogeneity across studies present considerable challenges to the synthesis of results in systematic reviews and meta-analyses (Smith et al., 2016). This can be addressed by future reviews when a higher number of studies are published on this particular subject matter.

## Conclusion

The present review found that parent-mediated interventions did not influence the ASD diagnosis in infants at risk of the disorder or reduce the risk of later ASD diagnosis. This is in line with earlier reviews with wider age ranges and type of interventions (Bradshaw et al., 2015; Debodinance et al., 2017). These interventions demonstrate the potential to enhance parental engagement, but evidence on effects on infants’ skills remains uncertain and further work would be needed before generalisations can be made. In the interests of future systematic reviews and meta-analyses, future studies need to enhance available evidence. Primarily, researchers are strongly encouraged to pursue consensus on the determinants of ASD risk, standardize outcome measures, and implement blinded researcher ratings to reduce bias. Longitudinal assessments, as implemented by five studies in this review (Baranek et al., 2015; Green et al., 2015; Jones et al., 2017; Kasari et al., 2014; Whitehouse et al., 2019), are essential to assess the long-term impacts of very early interventions. Later life skills such as language ability (Edmunds et al., 2019) may not have emerged when assessed at the end of studies.

Presently, we do not have definitive evidence that early parent-based interventions are useful in infants at risk for ASD but early interventions in ASD may provide children with a head start in life, as early life skills subsequently predict adult outcomes (Gillespie-Lynch et al., 2012). The programmes may need further refining, with families being engaged in the planning process (Pickard et al., 2017), and specific deficits being targeted by interventions. The American Academy of Pediatrics (AAP) recommends that all children would have been screened twice for developmental delays (at 9 and 18 months) and twice specifically for ASD (at 18 and 24 months) before the age of 2 (Centers for Disease Control & Prevention, 2020). The integration of autism assessments into routine developmental reviews are yet to be reflected in healthcare guidelines in the UK (National Institute for Health and Care Excellence, 2017) or Australia (Department of Health, 2013), where guidelines are in place only for children with identifiable regression or concerns. However, considerable evidence is needed from future studies to be able to recommend routine autism risk screening and the provision of high intensity interventions for infants under 2 years of age.

## Supplementary Information

Below is the link to the electronic supplementary material.

Supplementary file1 (DOC 66 kb)

Supplementary file2 (DOCX 16 kb)

## Footnotes

1 The authors recognise the need for less-stigmatising language in research. For the purposes of this systematic review, ‘Autism Spectrum Disorder (ASD)’ was used to align with the Diagnostic and Statistical Manual of Mental Disorders, 5^th^ edition (DSM-5) and all reviewed studies. This is also due to relevance and specific reference to interventions for infants who are more likely to be formally diagnosed with ASD.

## Declarations

### Research Involving Human and Animal Participants

This article does not contain any studies with human participants or animals performed by any of the authors.
