# Supplementary material for: Parent-Mediated Interventions for Infants under 24 Months at Risk for Autism Spectrum Disorder: A Systematic Review of Randomized Controlled Trials
Source: J Autism Dev Disord. 2021 Jul 8;52(6):2553–74. doi: 10.1007/s10803-021-05148-9 (PMC9114042; doi:10.1007/s10803-021-05148-9)
Supplement: Supplementary file 2 — Supplementary file2 (DOCX 16 kb) [file 10803_2021_5148_MOESM2_ESM.docx]

**Supplementary Material - Appendix**

Search terms and strategies implemented in electronic databases

**Ovid PsycINFO 1806 to October Week 4 2019 (N=165)**

1. interven*.mp. [mp=title, abstract, heading word, table of contents, key concepts, original title, tests & measures, mesh]

2. (infant* or baby or babies or "young child*" or toddler*).mp. [mp=title, abstract, heading word, table of contents, key concepts, original title, tests & measures, mesh]

3. (risk adj2 (autis* or asc or ASD or pdd or "pervasive developmental disorder*")).mp. [mp=title, abstract, heading word, table of contents, key concepts, original title, tests & measures, mesh]

4. 1 and 2 and 3

**Ovid PsycARTICLES Full Text (N=47)**

1. interven*.mp. [mp=title, abstract, full text, caption text]

2. (infant* or baby or babies or "young child*" or toddler*).mp. [mp=title, abstract, full text, caption text]

3. (risk adj2 (autis* or asc or ASD or pdd or "pervasive developmental disorder*")).mp. [mp=title, abstract, full text, caption text]

4. 1 and 2 and 3

**Ovid Global Health 1973 to 2019 Week 43 (N=6)**

1. interven*.mp. [mp=abstract, title, original title, broad terms, heading words, identifiers, cabicodes]

2. (infant* or baby or babies or "young child*" or toddler*).mp. [mp=abstract, title, original title, broad terms, heading words, identifiers, cabicodes]

3. (risk adj2 (autis* or asc or ASD or pdd or "pervasive developmental disorder*")).mp. [mp=abstract, title, original title, broad terms, heading words, identifiers, cabicodes]

4. 1 and 2 and 3

**Ovid MEDLINE(R) and Epub Ahead of Print, In-Process & Other Non-Indexed Citations and Daily 1946 to November 01, 2019 (N=131)**

1. interven*.mp. [mp=title, abstract, original title, name of substance word, subject heading word, floating sub-heading word, keyword heading word, organism supplementary concept word, protocol supplementary concept word, rare disease supplementary concept word, unique identifier, synonyms]

2. (infant* or baby or babies or "young child*" or toddler*).mp. [mp=title, abstract, original title, name of substance word, subject heading word, floating sub-heading word, keyword heading word, organism supplementary concept word, protocol supplementary concept word, rare disease supplementary concept word, unique identifier, synonyms]

3. (risk adj2 (autis* or asc or ASD or pdd or "pervasive developmental disorder*")).mp. [mp=title, abstract, original title, name of substance word, subject heading word, floating sub-heading word, keyword heading word, organism supplementary concept word, protocol supplementary concept word, rare disease supplementary concept word, unique identifier, synonyms]

4. 1 and 2 and 3

**Ovid EMBASE 1974 to 2019 Week 44 (N=161)**

1. interven*.mp. [mp=title, abstract, heading word, drug trade name, original title, device manufacturer, drug manufacturer, device trade name, keyword, floating subheading word, candidate term word]

2. (infant* or baby or babies or "young child*" or toddler*).mp. [mp=title, abstract, heading word, drug trade name, original title, device manufacturer, drug manufacturer, device trade name, keyword, floating subheading word, candidate term word]

3. (risk adj2 (autis* or asc or ASD or pdd or "pervasive developmental disorder*")).mp. [mp=title, abstract, heading word, drug trade name, original title, device manufacturer, drug manufacturer, device trade name, keyword, floating subheading word, candidate term word]

4. 1 and 2 and 3

**Web of Science Core Collection (N=180)**

1. (interven*)

*AND*

2. (infant* or baby or babies or "young child*" or toddler*)

*AND*

3. ((risk) near/2 (autis* or asc or ASD or pdd or “pervasive developmental disorder”))

**Proquest Dissertations & Theses Global (Senate House Library) (N=136)**

1. (interven*)

2. (infant* or baby or babies or "young child*" or toddler*)

3. ((risk) near/2 (autis* or asc or ASD or pdd or "pervasive developmental disorder"))

4. 1 AND 2 AND 3

**Figure Captions**

**Fig. 1** PRISMA flow diagram (Moher et al., 2009)

**Fig. 2** Assessment of risk of bias in studies based on Cochrane Collaboration’s tool (Higgins et al., 2011), with items D7 and D8 added for the purposes of this review. Risk of bias plot was created using *robvis* (McGuinness & Higgins, 2020)
